# Antidepressants strongly influence the relationship between depression and heart rate variability: findings from The Irish Longitudinal Study on Ageing (TILDA)

**DOI:** 10.1017/S0033291714001767

**Published:** 2014-07-30

**Authors:** C. O'Regan, R. A. Kenny, H. Cronin, C. Finucane, P. M. Kearney

**Affiliations:** 1 The Irish Longitudinal Study on Ageing (TILDA), Trinity College Dublin, Ireland; 2 University College Cork, Ireland

**Keywords:** Antidepressants, depression, heart rate variability, older adults

## Abstract

**Background:**

Heart rate variability (HRV) is known to be reduced in depression; however, is unclear whether this is a consequence of the disorder or due to antidepressant medication.

**Methods:**

We analysed data on 4750 participants from the first wave of The Irish Longitudinal Study on Ageing (TILDA). Time [standard deviation of normal to normal intervals (SDNN ms^2^)] and frequency domain [low frequency (LF) and high frequency (HF)] measures of HRV were derived from 3-lead surface electrocardiogram records obtained during 10 min of supine rest. Depression was assessed using the Center for Epidemiologic Studies – Depression scale.

**Results:**

Participants on antidepressants [with (*n* = 80) or without depression (*n* = 185)] differed significantly from controls (not depressed and not taking antidepressants *n* = 4107) on all measures of HRV. Depressed participants not taking antidepressants (*n* = 317) did not differ from controls on any measures of HRV. In linear regression analysis adjusted for relevant factors all antidepressants were associated with lower measures HRV. Participants on selective serotonin reuptake inhibitors (SSRIs) had higher measures of HRV relative to participants on tricyclic antidepressants or serotonin–norepinephrine reuptake inhibitors respectively.

**Conclusions:**

Our results suggest that reductions in HRV observed among depressed older adults are driven by the effects of antidepressant medications. SSRIs have less impact on HRV than other antidepressants but they are still associated with lower measures of HRV. Study limitations include the use of a self-report measure of depression and floor effects of age on HRV could have limited our ability to detect an association between HRV and depression.

## Introduction

Depression is a known risk factor for the development of cardiovascular disease (CVD) and an independent predictor of poor prognosis following a cardiac event (Lett *et al.* 2004). Alterations in the autonomic nervous system (ANS) including a reduction in heart rate variability (HRV) may partly explain the increased risk of CVD, since low HRV is a known risk factor for myocardial infarction, arrhythmias, and cardiac mortality (Tsuji *et al.* 1994; Dekker *et al.* 2000; Carney & Freedland, 2009).

Although there is strong evidence that HRV is reduced in depression, it remains unclear whether these reductions are due to the effects of antidepressant medication or the disease *per se*. Rottenberg (2007) summarized 13 studies (312 depressed patients and 374 controls) and found significantly reduced HRV in depression. Subsequently, a review by Kemp *et al.* (2010*b*) on 302 depressed patients who were free from CVD (424 controls) also demonstrated reductions in HRV among individuals with depression. By contrast, a large study by Licht *et al.* (2008) concluded that reductions in HRV among depressed participants were mainly driven by the effects of antidepressants. Importantly, they found that major depressive disorder (MDD) patients without antidepressant use (*n* = 1018) did not differ consistently from controls (*n* = 515) on measures of HRV. Moreover, longitudinal follow-up from this study confirmed that MDD was not associated with HRV and showed that MDD patients using antidepressant medication, particularly tricyclic antidepressants (TCAs) and serotonergic noradrenergic reuptake inhibitors (SNRIs) had significantly lower HRV than controls (Licht *et al.* 2010). It is notable that the sample size in Licht's study was far larger than the total number of participants in the previous meta-analyses. However, some methodological issues have been highlighted that hamper definitive conclusions (Kemp *et al*. 2010*a*). Adding to the debate, Brunoni *et al.* (2013) have suggested that decreased HRV may be a trait marker for depression and argue that the pathophysiological features of MDD, rather than pharmacotherapy drive the reported reductions in HRV. Their hypothesis is based on evidence from a study examining the effect of transcranial direct current stimulation (tDCS) and sertraline [a selective serotonin reuptake inhibitor (SSRI)] on HRV. Overall, depressed subjects were found to have lower HRV than controls; however, despite resolution of depressive symptoms, neither treatment was associated with changes in HRV.

Antidepressant treatment clearly impacts on HRV, although a precise picture has yet to emerge. A meta-analysis by Kemp *et al.* (2010*b*) found evidence that TCAs significantly reduce HRV but all other antidepressants had a benign effect on HRV. The influence of physical illness and lifestyle factors such as smoking, alcohol use, high body mass index (BMI), and low physical activity also needs to be considered given that these factors occur more frequently in depression and are associated with decreased HRV (Rosenwinkel *et al.* 2001; Friedman, 2007). Moreover, the need to examine the role of co-morbid anxiety in reducing HRV was recently highlighted by Kemp *et al.* (2012) who found that MDD patients with generalized anxiety disorder (GAD) had greater reductions in HRV compared to MDD patients without co-morbid anxiety and controls. GAD is the most prevalent anxiety disorder among older adults (Schoevers *et al.* 2003) and a high prevalence of co-morbid depression and anxiety is commonly observed in this age group (Lenze *et al.* 2000).

To date, most of the research on depression and HRV has been conducted on young and middle-aged patients with depression. Although valuable, it has been suggested that research on older adults who have a greater risk of heart disease could have more clinical relevance (Jindal *et al.* 2008). HRV is known to decrease across the lifespan (Jennings & Mack, 1984; Yeragani *et al.* 1997; Agelink *et al.* 2001) and consequently the impact of depression on HRV is also likely to vary across age cohorts. Age-related decline may lower HRV to levels associated with increased risk of mortality therefore distinguishing low HRV due to depression from that due to normal ageing is challenging. It is possible that depression in old age may further reduce age-related declines in HRV and exacerbate the risk of cardiovascular morbidity (Gehi *et al.* 2005). Moreover, depression that first presents in late life is believed to have a different aetiopathogenesis from earlier-onset depression and this difference in pathophysiology may have specific consequences for autonomic function in older adults with depression.

Of the few studies that have investigated HRV in depressed older adults the results are conflicting; with some studies providing evidence of reduced HRV in depression (Carney & Freedland, 2009; Dauphinot *et al.* 2012) and others reporting no association (Gehi *et al.* 2005; Jindal *et al.* 2008). Notably, none of these studies have investigated the role of individual antidepressant classes on HRV; therefore, in older adults their effects on HRV are largely unknown. The aim of this study is to examine whether depression is associated with reduced HRV in older adults and investigate the extent to which any associations observed are confounded by lifestyle, co-morbid anxiety or the effect of antidepressant medication.

## Methods

### Study design and participants

We analysed data from the first wave of The Irish Longitudinal Study on Ageing (TILDA) collected between October 2009 and February 2011. Full details of the sampling procedure and response rate have been described elsewhere (Kearney *et al.* 2011*a*). In brief, TILDA is a nationally representative study of people aged ⩾50 years resident in Ireland. People with known or suspected dementia were ineligible at baseline for participation in TILDA.

Participants completed a computer-assisted personal interview (CAPI) in their own homes administered by trained professional interviewers. The TILDA questionnaire includes detailed questions on health, social and financial circumstances. Each participant was then invited to travel to one of two health centres for a comprehensive health assessment. Participants who were unable or unwilling to attend a health centre were offered a modified assessment in their own home. All health assessments were carried out by trained nurses. The study was approved by the Faculty of Health Sciences Research Ethics Committee at Trinity College Dublin, and participants were required to provide written informed consent prior to participation in the study. The measures specific to the current analysis are described in detail below.

## Ethics

The authors assert that all procedures contributing to this work comply with the ethical standards of the relevant national and institutional committees on human experimentation and with the Helsinki Declaration of 1975, as revised in 2008.

### Psychiatric assessment

Depression was assessed using the Center for Epidemiologic Studies – Depression scale (CES-D). The CES-D generates a total score with a range between 0 and 60 with higher scores indicating greater depressive symptoms. A cut-off score of 16 has been shown to have a sensitivity of 100% and specificity of 88% for MDD in an elderly population (Beekman *et al.* 1997).

Anxiety was assessed using the Hospital Anxiety Depression Scale – Anxiety subscale (HADS-A). Scores from this 7-item scale range from 0 to 21 with higher scores indicating greater anxiety symptoms. A cut-off score of ⩾11 has been used to classify participants with clinically significant anxiety (Zigmond & Snaith, 1983).

### Measurement of HRV

HRV was only assessed during the health centre assessment. A continuous 10 min supine resting surface 3-lead electrocardiogram (ECG) was digitally recorded using the Medilog AR12 system (Schiller, Switzerland). Each recording was conducted in a comfortably lit, quiet room at ambient temperature (21–23°C). Subjects were instructed to breath spontaneously for the first 5 min period, and to control their breathing (paced) during the second 5 min period according to a pre-recorded set of auditory instructions (set at a rate of 12 breaths/min). Paced breathing experimentally controlled for the effect of respiratory rate on spectral HRV indices (Sandercock *et al.* 2008). The acquired ECG was sampled at 4 kHz, band-pass filtered and a proprietary algorithm was used to detect the R peak of each heart beat recorded on the ECG signal (Pardey & Jouravleva, 2004). Supra-ventricular ectopic beats and noise were excluded from the signal using linear interpolation. All recordings were screened for atrial fibrillation (AF) using criteria from the European Society of Cardiology (Camm *et al.* 2010), and those identified with AF were subsequently excluded from analysis. Mean resting heart rate (HR) was calculated over 5 min of spontaneous breathing.

Time domain measures derived from each 5 min epoch included the standard deviation of normal to normal intervals (SDNN ms^2^). Frequency domain (FD) features were calculated from spectral estimates derived using an autoregressive (Burg transform) parametric algorithm. FD features were derived by integrating the power spectral density across two frequency bands: *low frequency power* (LF, 0.04–0.15 Hz, ms^2^) and *high frequency power* (HF, 0.15–0.4 Hz, ms^2^). HF measures are thought to reflect parasympathetic activity while LF measures are thought to reflect both sympathetic and parasympathetic activity.

### Measurement of covariates

Sociodemographic characteristics included age, sex, and highest level of educational attainment [primary (<8 years), secondary (8–12 years), tertiary (⩾12)]. In addition, the following health indicators were considered as covariates as these have been linked with both depression status and HRV. Objective measures of weight (one measure using SECA electronic floor scales) and height (one measure using SECA 240 wall-mounted measuring rod) were used to calculate BMI. Physical activity was assessed using the International Physical Activity Questionnaire – Short Form (Craig *et al.* 2003). Participants were classified into three groups representing low, medium and high levels of exercise. Participants self-reported how many standard alcoholic drinks they consumed in a week as well as whether they were a non-smoker, former smoker or current smoker. Self-reports were used to ascertain the presence of a doctors diagnosis of heart disease and other chronic conditions. Blood pressure (BP) was recorded using an automated oscillometric BP monitor (Omron model M10-IT). Participants were classified as hypertensive if the mean of their two seated systolic blood pressure measurements was ⩾140 mmHg and/or if the mean of their two seated diastolic blood pressure measurements was ⩾90 mmHg (Mancia *et al.* 2007).

Medication use was determined by recording medication names from the medicine bottles in the participant's home. Medications were classified using the World Health Organization Anatomical Therapeutic Chemical (ATC) system (WHO, 2011). A dichotomous variable for cardioactive medication was computed (1=yes, 0=no) with the following ATC codes: antihypertensive drugs, C02; diuretic drugs, C03; peripheral vasodilator drugs, C04; vasoprotective drugs, C05; *β*-blocking agents, C07; and calcium-channel blockers, C08. Dichotomous variables (1=yes, 0=no) for various classes of antidepressant medications were computed with the following ATC codes: SSRIs, N06AB; TCAs, N06AA; monoamine oxidase inhibitors (MAOIs), N06AF; SNRIs, NO6AX (16, 17, 23, 21); serotonin antagonist + reuptake inhibitors, N06AX (05, 06). We also distinguished benzodiazepine drugs (ATC codes N03AE, N05BA, N05CD, N05CF) and other non-depression-related psychoactive medication such as anaesthetic drugs, ATC code N01; analgesic drugs, ATC code N02; antiepileptic drugs, ATC code N03; anti-Parkinson disease drugs, ATC code N04; psycholeptic drugs, ATC code N05; psychostimulants, ATC code N06B; antidementia drugs, ATC code N06D; and other nervous system drugs, ATC code N07.

### Statistical analysis

Statistical analysis was performed using SPSS version 20 (IBM Corp, USA). Distribution of continuous variables was assessed using Q-Q plots and histograms. Non-normally distributed measures of HRV (SDNN, LF, HF) were log-transformed for graphs and analysis (values were back-transformed for presentation of regression analysis and between group comparisons of adjusted means but not for graphs). Normally distributed variables were described as means and standard errors (s.e.), and were compared using independent *t* tests and categorical variables were compared using *χ*^2^ tests.

Baseline clinical and demographic characteristics among groups were assessed using ANOVA or the *χ*^2^ statistics. Linear regression was used to calculate mean values of HR, SDNN, LF and HF adjusted for age, sex, education, BMI, smoking, alcohol use, physical activity, heart disease, cardioactive medications and number of chronic diseases for each depression group. In an attempt to understand the individual effects of various types of psychoactive medication on HRV, we distinguished between participants on various types of psychoactive medication (exclusively) and compared their mean HR and HRV (raw values) with unmedicated controls.

Subsequently, the effect of anxiety and psychoactive medication on the relationship between depression and HRV was examined by entering information on these variables into a number of regression models with adjustment for potential confounders including age, sex, education, physical activity, alcohol use, number of chronic conditions (diabetes, arthritis, cancer, chronic lung disease, liver disease, stomach ulcers, substance abuse), history of CVD (angina, stroke, myocardial infarction), smoking (0=non/previous smoker, 1=current smoker), BMI and antihypertensive drugs as covariates. Unstandardized regression coefficients with 95% confidence intervals (CIs) and significance levels are presented here. Differences with *p* < 0.05 (two-tailed) were considered statistically significant.

## Results

At baseline 8175 participants were recruited to the study. The household response rate was 62%. In total, 5034 (61%) participants who completed a health centre assessment were eligible for inclusion. Of these 96% successfully completed the CES-D and measurements of HRV, giving the 4750 included in the current analysis. The mean age was 61.6 years (s.d. = 8.3 years) and 55% of participants were female.

To test whether measures of HRV differed across persons with and without depression and according to antidepressant use, four distinct depression groups were created for the present study: 4107 controls without depression (score <16 on CES-D scale) or antidepressant use; 185 individuals without depression (score <16 on CES-D scale) but currently on antidepressants which we labelled as the ‘remitted’ group; 317 individuals with depression but not currently on antidepressants; and 80 individuals with depression and currently on antidepressants. Table 1 presents demographic characteristics, disease status, lifestyle behaviours, and medication use according to depression groups. Mean CES-D score was similarly high in both groups with depression (22 in participants not taking antidepressants and 25 in those on antidepressants). Relative to controls, participants with depression (current or remitted) were more likely to be female, had less education, were less physically active, were more likely to smoke and had more heart and other chronic diseases. Additionally, they had much higher levels of anxiety and medication use other than antidepressants (benzodiazepines, heart and blood pressure medications use and other psychotropic medications).

**Table 1.** Sample characteristics by depression group

	Non-depressed controls (<16 on CES-D & no antidepressants)	Remitted depression (<16 on CES-D & on antidepressants)	Current depression (⩾16 on CES-D & no antidepressants)	Current depression (⩾16 on CES-D & on antidepressants)	*p* value
	(*N* = 4107)	(*N* = 185)	(*N* = 317)	(*N* = 80)	
Age, mean (s.e.)	61.7 (0.13)	62.6 (0.62)	60.4 (0.43)	59.4 (0.75)	**0.001**
Female sex	2179 (53.1)	124 (67.0)	212 (66.9)	58 (72.5)	**<0.001**
Education					
Primary	828 (20.2)	46 (24.9)	91 (28.7)	27 (33.8)	**<0.001**
Secondary	1743 (42.5)	80 (43.2)	117 (36.9)	33 (41.3)	0.279
Tertiary	1534 (37.4)	59 (31.9)	109 (34.4)	20 (25.0)	**0.045**
BMI^a^, mean (s.e.)	28.5 (0.08)	28.8 (0.41)	28.6 (0.29)	29.3 (73)	0.415
Physical activity^b^					
Low	1045 (25.6)	68 (37.0)	123 (39.0)	34 (42.5)	**<0.001**
Medium	1501 (36.8)	63 (34.2)	106 (33.7)	26 (32.5)	0.522
High	1529 (37.5)	53 (28.8)	86 (27.3)	20 (25.0)	**<0.001**
Smoking status					
Never	1948 (47.4)	72 (38.9)	119 (37.5)	24 (30.0)	**<0.001**
Former	1600 (39.0)	75 (40.5)	110 (34.7)	35 (43.8)	0.341
Current	559 (13.6)	38 (20.5)	88 (27.8)	21 (26.3)	**<0.001**
Alcohol^c^	5.98 (0.15)	6.10 (0.78)	5.25 (0.55)	6.95 (2.2)	0.493
Heart disease^d^	443 (10.8)	30 (16.2)	45 (14.2)	16 (20.0)	**0.003**
No chronic conditions^e^	0.45 (0.01)	0.84 (0.06)	0.68 (0.04)	0.98 (0.10)	**<0.001**
Anxiety^f^, mean (s.e.)	5 (0.07)	6 (0.38)	9 (0.34)	11 (0.75)	<0.001
Severity of depression (mean CES-D score)	4 (0.08)	6 (0.43)	22 (0.52)	25 (1.3)	<0.001
Medication use					
Selective serotonin reuptake inhibitors		102 (55.1)		49 (61.2)	0.463
Serotonin–norepinephrine reuptake inhibitors		36 (19.5)		17 (21.2)	0.919
Serotonin antagonist + reuptake inhibitors		1 (0.5)		1 (1.3)	0.540
Tricyclic antidepressants		50 (27.0)		21 (26.3)	0.896
Monoamine oxidase inhibitors		1 (0.8)		0	0.510
Benzodiazepines	115 (2.8)	43 (23.2)	44 (13.9)	26 (32.5)	**<0.001**
Other psychotropic medication	244 (5.9)	42 (22.7)	59 (18.6)	20 (25.0)	**<0.001**
Heart or blood pressure medication	1303 (31.7)	77 (41.6)	97 (30.6)	34 (42.5)	**0.007**

CES-D, Center for Epidemiologic Studies – Depression scale; BMI, body mass index.

a Weight in kilograms divided by height in metres squared.

b Exercise in three groups reported according the International Physical Activity Questionnaire (short form) scoring protocol.

c Standard number of drinks a week (standard drinks a day by weekly frequency).

d Angina or myocardial infarction or heart failure or abnormal heart rhythm.

e Number of chronic conditions (diabetes, stroke, arthritis, cancer, stomach ulcers, substance abuse or liver disease.

f Mean score from 7-item Hospital Anxiety and Depression Scale – anxiety.

**Fig. 1.**
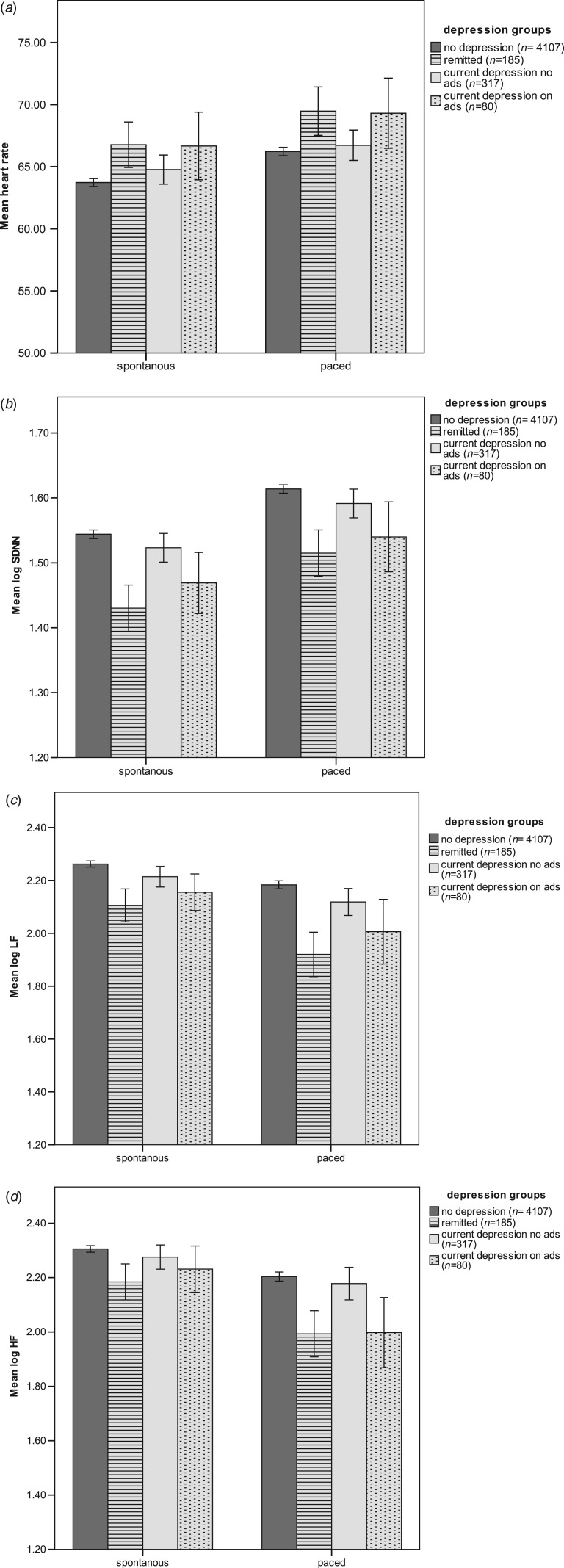
Mean values for spontaneous and paced breathing conditions across depression groups (raw data) for (*a*) heart rate, (*b*) log standard deviation of normal to normal intervals (SDNN), (*c*) log low frequency (LF), (*d*) log high frequency (HF). Error bars: 95% confidence intervals.

Fig. 1 shows mean raw log values of HR, SDNN, LF and HF during spontaneous and paced breathing conditions for the four depression groups. As illustrated, HR is increased in participants taking antidepressants [with (paced mean=69.3) or without (paced mean=69.5) depression] compared to controls (paced mean=66.2) or participants with depression who were not taking antidepressants (paced mean=66.7). A similar pattern emerges for measures of SDNN, LF and HF whereby depressed participants not taking antidepressants (e.g. paced mean SDNN = 1.59) have similar measures of HRV to controls (e.g. paced mean SDNN = 1.61); and participants taking antidepressants [with (e.g. paced mean SDNN = 1.54) or without depression (e.g. paced mean SDNN = 1.52)] have significantly lower measures of HRV than controls. For conciseness, only measures of HRV obtained from the paced breathing condition (5 min) are presented in the subsequent analysis, since the pattern of findings was similar in both breathing conditions.

Table 2 presents unadjusted and adjusted means (back transformed) for HR, SDNN, LF and HF across depression groups. Depressed participants who were not currently taking antidepressants did not differ significantly from controls on adjusted measures of SDNN, LF or HF. Participants who were on antidepressants (with or without depression) differed significantly from controls on all measures of HRV. In general, the ‘remitted’ group had the lowest measures of HRV observed in our study.

**Table 2.** HR, SDNN intervals, LF, and HF by depression group during the paced breathing condition

	Non-depressed controls^a^ (<16 on CES-D & no antidepressants)	Remitted depression^b^ (<16 on CES-D & on antidepressants)	Current depression^c^ (⩾16 on CES-D & no antidepressants)	Current depression^d^ (⩾16 on CES-D & on antidepressants)	Controls *v.*					
	(*N* = 4107)	(*N* = 185)	(*N* = 317)	(*N* = 80)	Remitted (<16 on CES-D and on antidepressants)		Current depression (no antidepressants)		Current depression (on antidepressants)	
	Mean (s.e.)	Mean (s.e.)	Mean (s.e.)	Mean (s.e.)	Effect size^b^	*p* value	Effect size^b^	*p* value	Effect size^b^	*p* value
	**HR (beats/min)**									
Unadjusted	66.2 (0.17)	69.4 (0.98)	66.7 (0.61)	69.3 (1.4)	−3.2	0.00**	−0.79	0.42	−2.4	0.01**
Adjusted^a^	66.3 (0.03)	66.7 (0.16)	67.1 (0.13)	67.3 (0.26)	2.0	0.04*	3.9	0.00**	2.7	0.01**
	**SDNN**^c^									
Unadjusted	40.7 (1.0)	32.3 (1.0)	38.9 (1.0)	34.6 (1.1)	4.5	0.00**	2.2	0.06	2.2	02*
Adjusted^a^	40.7 (1.0)	38.9 (1.0)	39.8 (1.0)	39.7 (1.0)	−6.11	0.00**	−1.1	0.25	−2.0	0.04*
	**LF**^d^ **(ms^2^)**									
Unadjusted	151.3 (1.0)	83.1 (1.1)	128.8 (1.1)	102.3 (1.1)	5.0	0.00**	1.7	0.02*	3.4	0.00**
Adjusted^a^	147.9 (1.0)	128.8 (1.0)	144.5 (1.0)	134.8 (1.1)	−8.5	0.00**	−1.3	0.18	−2.5	0.01**
	**HF**^e^ **(ms^2^)**									
Unadjusted	158.4 (1.0)	97.7 (1.1)	147.9 (1.1)	97.7 (1.2)	2.4	00**	1.0	0.41	2.4	0.01**
Adjusted^a^	158.8 (1.0)	147.2 (1.0)	155.0 (1.0)	144.8 (1.1)	−4.8	0.00**	1.0	0.31	2.3	0.02*

HR, Heart rate; SDNN, standard deviation of normal to normal intervals (SDNN); LF, low frequency; HF, high frequency; CES-D, Center for Epidemiologic Studies – Depression scale.

a Adjusted for age, sex, education, body mass index, physical activity, smoking, alcohol use, heart disease, chronic disease, heart medication, anxiety.

b *t* test used for unadjusted means, *z* test used for adjusted means.

* Denotes significance at 0.05.

** Denotes significance at 0.01.

Six mutually exclusive medications groups were created to examine the individual effects of various types of psychoactive medication on measures of HRV: 3778 controls who were not taking antidepressants; 91 individuals who were only taking SSRI antidepressants; 34 individuals who were only taking TCAs; 31 individuals who were only taking SNRIs; 119 individuals who were only taking benzodiazepines and 263 participants who were taking medications collapsed into the group labelled ‘other psychotropic medications’.

**Fig. 2.**
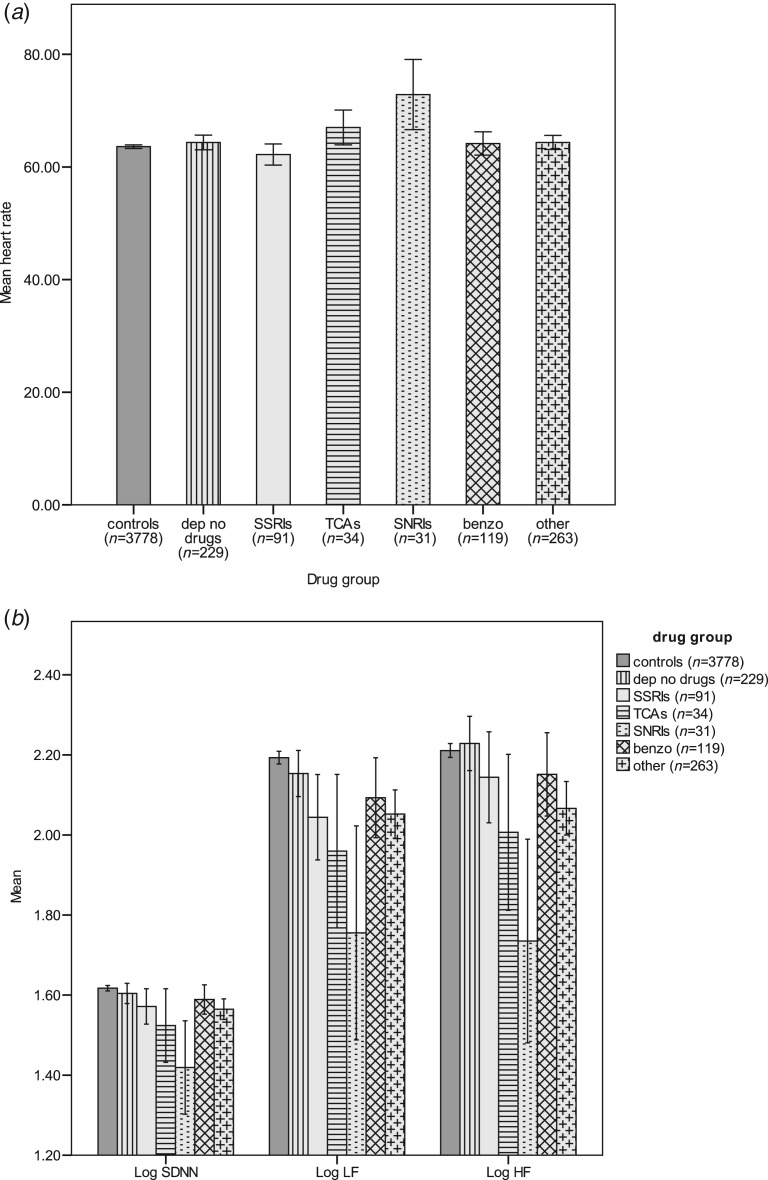
Mean values by depression and medication group for (*a*) heart rate, (*b*) log standard deviation of normal to normal intervals (SDNN), low frequency (LF) and high frequency (HF) (raw data). Error bars: 95% confidence intervals.

Fig. 2*a, b* shows raw log values of HR, SDNN, LF and HF for each of these groups. Relative to controls, mean heart rates are significantly higher (*p* < 0.001) in participants on SNRIs and TCAs; whereas mean heart rates in participants on SSRIs are similar to controls. Once more, a similar pattern emerged for the effect of antidepressant medication on indices of HRV; whereby a graded decrease in HRV, dependent on antidepressant type was observed among participants. Almost all measures of HRV were significantly lower in participants on antidepressants. Participants on SNRIs had the lowest measures of HRV observed in our study. Among participants on antidepressant medication, SSRIs were associated with the highest measures of HRV. Participants on benzodiazepines did not differ from controls on measures of HRV; however, participants using ‘other psychoactive medication’ had significantly lower measures of HRV relative to controls.

Table 3 presents linear regression models examining the relationship between depression and HRV and the role of anxiety and psychoactive medication. In univariate analysis (model A) anxiety and all antidepressants are associated with increased HR; however, associations persist only for TCAs and SNRIs in the fully adjusted model (model C). Depression and all antidepressants are associated with reduced SDNN in univariate analysis; however, in the fully adjusted model only antidepressants (SSRIs, TCAs, SNRIs) and ‘other psychotropic’ medications are associated with significantly lower measures of SDNN. Finally, in fully adjusted analyses, SNRIs are associated with significantly lower measures of LF and both TCAs and SNRIs are associated with significantly lower measures of HF.

**Table 3.** Linear regression models of depression, anxiety and medications on HR and measures of HRV

	Model A^a^	Model B^b^	Model C^c^
	*b* (95% CI)	*b* (95% CI)	*b* (95% CI)
HR			
Depression	**0.886** (−0.246 to 2.01)	**0.034** (−1.26 to 1.33)	**0.310** (−1.66 to 1.04)
Anxiety	1.33* (0.175 to 2.50)	**0.736** (−0.495 to 2.02)	**0.666** (−0.627 to 1.96)
SSRIs	**−0.373** (−2.17 to 1.43)	**−0.405** (−2.42 to 1.61)	**−0.717** (−2.82 to 1.39)
TCAs	5.04** (2.39 to 7.70)	3.78** (0.918 to 6.64)	3.54** (0.546 to 6.53)
SNRIs	10.07** (7.10 to 13.0)	11.1** (8.01 to 14.2)	11.5** (8.35 to 14.7)
Benzodiazepines	**1.29** (−0.170 to 2.75)	**0.419** (−1.25 to 2.09)	**0.525** (−1.20 to 2.26)
Other psychotics	**1.04** (−0.137 to 2.22)	**0.574** (−0.710 to 1.85)	**0.368** (−0.991 to 1.72)
Log SDNN			
Depression	−1.06** (−1.12 to −1.01)	**−1.04** (−0.1.11 to 1.01)	**−1.03** (−1.09 to 1.02)
Anxiety	**1.00** (−1.04 to 1.06)	**1.04** (−1.00 to 1.10)	**1.01** (−1.03 to 1.07)
SSRIs	−1.16** (−1.26 to −1.07)	−1.12** (−1.23 to −1.03)	−1.09* (−1.20 to −1.00)
TCAs	−1.03** (−1.42 to −1.12)	−1.17** (−1.33 to −1.03)	−1.18* (−1.35 to −1.03)
SNRIs	−1.42** (−1.62 to −1.24)	−1.44** (−1.66 to −1.25)	−1.51** (−1.73 to −1.58)
Benzodiazepines	−1.10** (−1.17 to −1.03)	**−1.00** (−1.08 to 1.06)	**−1.01** (−1.05 to 1.09)
Other psychotics	−1.15** (−1.21 to −1.09)	−1.11** (−1.18 to −1.05)	−1.09** (−1.16 to −1.03)
Log LF			
Depression	−1.19** (−1.34 to −1.05)	−1.14* (−1.30 to −1.00)	**−1.11** (−1.28 to 1.02)
Anxiety	**1.03** (−1.08 to 1.17)	**1.19** (1.05 to 1.36)	**1.09** (−1.04 to 1.24)
SSRIs	−1.50**(−1.81 to −1.24)	−1.35**(−1.67 to −1.10)	**−1.22** (−1.51 to 1.10)
TCAs	−1.71** (−2.25 to −1.29)	−1.38* (−1.87 to −1.03)	**−1.30** (−1.76 to 1.03)
SNRIs	−2.50** (−3.41 to −1.83)	−2.67** (−3.71 to −1.93)	−2.86** (−3.95 to −2.07)
Benzodiazepines	−1.33** (−1.55 to −1.14)	**−1.03** (−1.56 to −1.19)	**1.08** (−1.09 to 1.29)
Other psychotics	−1.47** (−1.67 to −1.30)	−1.36** (−1.36 to −1.19)	−1.29** (−1.48 to −1.12)
Log HF			
Depression	**−1.12** (−1.28 to 1.00)	**−1.04** (−1.20 to 1.11)	**−1.05** (−1.23 to 1.09)
Anxiety	**1.05** (−1.20 to 1.08)	**1.04** (−1.10 to 1.20)	**−1.12** (−1.30 to 1.02)
SSRIs	−1.29* (−1.59 to −1.05)	**−1.19** (−1.50 to 1.05)	**−1.13** (−1.43 to 1.11)
TCAs	−1.67** (−2.26 to −1.23)	**−1.36** (−1.89 to 1.02)	−1.42* (−1.69 to −1.01)
SNRIs	−2.79** (−3.92 to −1.69)	−3.01** (−4.31 to −2.10)	−3.22** (−4.06 to −2.25)
Benzodiazepines	−1.19* (−1.41 to −1.01)	**1.03** (−1.17 to 1.25)	**1.10** (−1.09 to 1.34)
Other psychotics	−1.45** (−1.66 to −1.27)	−1.37** (−1.59 to −5.62)	−1.37** (−1.59 to −1.18)

HR, Heart rate; HRV, heart rate variability; SDNN, standard deviation of normal to normal intervals; LF, low frequency; HF, high frequency; CI, confidence interval; SSRIs, selective serotonin reuptake inhibitors; TCAs, tricyclic antidepressants; SNRIs, serotonin–norepinephrine reuptake inhibitors.

a Univariate analysis of depression, anxiety and medications on HR and measures of HRV.

b Adjusted for depression, anxiety and antidepressants.

c Model B additionally adjusted for age, sex, education, body mass index, physical activity, smoking, alcohol use, heart disease, number of chronic conditions, heart medications.

## Discussion

HRV in depression is now an important issue since both depression and decreased HRV have been shown to be predictors of cardiac morbidity and mortality. As a result, there has been substantial interest in the relationship between HRV and depression and the role, if any, of antidepressant medications. Our study has shown that antidepressants, rather than depression, have a significant impact on HR and measures of HRV. In older adults there exists a paucity of studies examining the effects of antidepressants on HRV, despite the high prevalence of CVD and increased prescribing of antidepressants in this age group.

Co-morbid anxiety or lifestyle factors did not explain our results. Overall, participants taking antidepressants (with or without current depression) had significantly lower measures of HRV than controls; whereas depressed individuals not taking antidepressants did not differ significantly from controls on all measures of HRV. This suggests that antidepressants rather than depression are responsible for the lower measures of HRV observed in our participants. If depression was responsible, we might have expected the remitted group to have superior measures of HRV compared to participants who were currently depressed but not taking antidepressants, particularly given some evidence that suggests antidepressants may reverse reductions in HRV associated with depression (Lavretsky *et al.* 1998; Touyz, 2005). This is not what we observed, in fact; the remitted group had the lowest measures of HRV observed in our study. Of course it must be acknowledged that some participants in our remitted group may have been taking antidepressants for conditions other than depression (anxiety, sleep problems, pain) and this could have impacted our findings, particularly the absence of an independent effect depression on HRV. Nevertheless, our results imply that antidepressants are associated with lower HRV in older adults. It has recently been hypothesised that low HRV may be a ‘trait’ rather that ‘state’ marker of depression (Brunoni *et al.* 2013). Our findings do not provide strong support for this hypothesis in older adults, since participants with depression who were not taking antidepressants had measures of HR and HRV that were in most cases similar to controls and in regression analysis depression was not associated with significantly lower HRV once antidepressants were considered in the model.

In our study, we compared measures of HRV between controls and participants who were only taking one class of psychoactive medication. This allowed us to examine the effect of individual medication classes on HRV. Overall, we found that participants on antidepressants (SSRIs, TCAs, SNRIs) had lower measures of HRV relative to controls; and those exclusively on SNRIs had the lowest measures of HRV recorded in our study. Although participants on SSRIs have better indices of HRV compared to participants on other antidepressants (TCAs and SNRIs), their measures are still significantly lower that controls on SDNN (*p* = 0.051) and LF (*p* = 0.007). The difference was non-significant (*p* = 0.251) for the HF (parasympathetic) measure of HRV. In SSRI users, low HRV was not accompanied by increased HR, providing support for the proposition (Licht *et al.* 2010) that SSRI's may have a beneficial effects on sympathetic activity that could counter their negative effects on parasympathetic activity and protect against CVD (Licht *et al.* 2010).

Overall, our results imply that HRV is not reduced in older adults with depression. In univariate analysis, depression was associated with reduced HRV; however, the association lost significance once antidepressant medications were included in the model. All antidepressants were associated with reduced measures of SDNN – our overall measure of HRV. SNRIs and TCAs were associated with reduced HF (parasympathetic) while only SNRIs were associated with significantly reduced LF (predominantly sympathetic). As a result, the hypothesis that reductions in HRV observed in depression are driven by the effects of antidepressants (Licht *et al.* 2008) is supported by our findings. HRV functioning is strongly age dependent, so our inferences must be constrained to older adults since the impact of depression on HRV may vary across age cohorts. In light of this, the findings of Kemp *et al*. (2010*b*, 2012) although in conflict with the findings of our study may also be true and reflect a differential relationship between depression and HRV in younger aged adults. In addition, floor effects of age on HRV could have limited our ability to detect reduced HRV due to depression from that due to normal aging and in this context our significant findings relating to antidepressants and HRV do appear robust.

The mechanisms through which antidepressants exert their effects on sympathetic/parasympathetic control over the heart remain incompletely understood. Increases in HR and decreases in HRV are believed to be due, at least in part, to the degree of anticholinergic activity associated with different antidepressant medications leading to diminished cardiac vagal tone (Lavretsky *et al.* 1998). At the brainstem level, serotonin reuptake inhibition may influence various relay nuclei of the parasympathetic nervous system (Raul, 2003; Ramage & Villalon, 2008). The antivagal effects of TCAs and SNRIs are believed to occur largely in the heart itself. Both types of antidepressants inhibit the reuptake of norepinephrine, causing a major increase in norepinephrine in the synaptic cleft (Esler *et al.* 1985).

Although our findings suggest that depression is not associated with impaired HRV we must acknowledge that our measure of depression was not a formal diagnosis of MDD and this may have influenced our findings. A formal classification of MDD may have revealed an association between depression and HRV which persisted after controlling for antidepressant use. Previous evidence of a negative association between depression severity and HRV, such that the more severe the depression, the lower the HRV provides support for this possibility (Kemp *et al.* 2010*a*, *b*). Accordingly, our analyses might have missed important depression effects if the cut-off score did not discriminate correctly. We therefore repeated the regression analysis using the CES-D as a continuous measure and found a similar pattern of findings to those using a cut-off score. Subsequent waves of the TILDA study include the DSM-IV based Composite International Diagnostic Interview – short form which will allow us to test the validity of our findings compared with a gold standard measure of depression.

We must also acknowledge that antidepressant use could be a marker of disease severity; therefore depression and not the antidepressants could have caused the reductions in HRV observed in our study. However, depression is known to be under-diagnosed and under-treated in older adults and estimates suggest that less than 20% of cases are detected or treated in the community (Cole & Dendukuri, 2003). We therefore would expect the group representing participants who were classified as depressed by the CES-D but who were not taking antidepressants to have included individuals with undiagnosed MDD. If the disorder and not the antidepressants were responsible for impaired HRV we might have expected to observe lower measures of HRV in this group. However, this was not the case; depressed participants not taking antidepressants had measures of HRV that were similar to controls. A further consideration is that our analysis assumes that depressed individuals not taking antidepressants are similar to depressed individuals who take antidepressants and this may not be correct. Prior to treatment, depression severity could have been greater in those taking antidepressants, so longitudinal data are required to disentangle whether the observed effects are due to some intrinsic effects of severe depression or an independent antidepressant effect. HRV was only assessed in participants who attended for a centre based assessment. Non-responders to the health centre assessment were older than health centre attendees; but importantly, were not more severely depressed. Although, mean CES-D score was slightly higher in participants who did not have a health assessment (6.2) and those who opted for a home assessment (6.8) compared to those who attended a health centre (5.6), these differences were not statistically significant (*p* = 0.084).

The main strengths of this study are the large population representative sample and comprehensive health assessment. Our paced breathing condition controlled for respiration rate, indicating that the relationships we observed are independent of variations in respiratory sinus arrhythmia driven by respiration. Limitations include that the data was analysed cross-sectionally and thus causal inferences cannot be made. Although many relevant risk factors were assessed and statistically controlled for in the analysis, the possibility of residual confounding exists. Participants were not randomly allocated to our groups so we acknowledge that attempts to statistically correct for group differences (as in Table 2) are contentious. However, our primary conclusions are drawn from the regression analyses presented in Table 3 and supported by the patterns observed in the graphs of raw data along with the comparisons of adjusted means from Table 2. Participants were not requested to refrain from eating, smoking, alcohol, caffeine, exercise, or medications prior to assessment, and time of day for assessment was not restricted based on practicality of delivering health assessments to participants from all over the country (Kearney *et al.* 2011*b*). These factors may affect reproducibility of results; however, time of day and food ingestion did not influence orthostatic blood pressure behaviour (another marker of autonomic function) in a previous TILDA pilot study (Fan *et al.* 2012).

In summary, this study provides an important clarification of the impact of depression and antidepressant medication on HRV. Our findings indicate that while SSRIs have less impact on HRV than other antidepressants with anticholinergic effects; they are still associated with lower measures of HRV. In older adults, the use of SNRIs poses a particular risk to the cardiovascular system since they are associated with the lowest measures of HRV observed in our study. Given that antidepressants such as SSRIs are now prescribed not only for depression, but also for a wide range of conditions; this issue has relevance to the general population. Longitudinal evidence is required to determine whether observed effects of antidepressants on HRV translate into CVD morbidity and mortality in depressed older adults.
